# Trends in the dispensing of opioids for pain and concurrent benzodiazepine use among First Nations People in Ontario, Canada, from 2013 to 2021

**DOI:** 10.17269/s41997-025-01097-3

**Published:** 2025-08-18

**Authors:** Alice Holton, Tianru Wang, Bisola Hamzat, Sacha Bragg, Bernadette deGonzague, Graham Mecredy, Tonya Campbell, Tony Antoniou, Lorrilee McGregor, Jonathan Bertram, Tara Gomes

**Affiliations:** 1 Li Ka Shing Knowledge Institute, St. Michael’s Hospital, Toronto, ON Canada; 2 MAP Centre for Urban Health Solutions, St. Michael’s Hospital, Toronto, ON Canada; 3 RCSI School of Pharmacy and Biomolecular Sciences, Dublin, Ireland; 4 ICES, Toronto, ON Canada; 5 Chiefs of Ontario, Toronto, ON Canada; 6 Department of Family and Community Medicine, Unity Health, Toronto, ON Canada; 7 Department of Family and Community Medicine, University of Toronto, Toronto, ON Canada; 8 Northern Ontario School of Medicine University, Sudbury, ON Canada; 9 Centre for Addiction and Mental Health, Toronto, ON Canada; 10 Leslie Dan Faculty of Pharmacy, University of Toronto, Toronto, ON Canada; 11 Institute for Health Policy, Management and Evaluation, University of Toronto, Toronto, ON Canada

**Keywords:** First Nations, Opioids for pain, Benzodiazepines, Concurrent use, Premières Nations, Opioïdes contre la douleur, Benzodiazépines, Emploi concomitant

## Abstract

**Objectives:**

To investigate dispensing trends and the characteristics of First Nations People in Ontario dispensed an opioid for pain and concurrent benzodiazepine treatment.

**Methods:**

We conducted a population-based serial cross-sectional study by quarter of registered (Status) First Nations People in Ontario who were dispensed an opioid for pain between April 1, 2013, and December 31, 2021. We reported quarterly trends in prevalent and incident opioid dispensing (rates per 1000 people), and the prevalence of concurrent benzodiazepine use among individuals receiving opioids for pain. For the final year (2021), we stratified rates by age, place of residence (within or outside First Nations communities), and sex.

**Results:**

Between 2013 and 2021, the quarterly rate of opioid dispensing for pain decreased by 25.0% among First Nations People in Ontario, from 74.7 to 56.0 per 1000 people. In stratified analyses for the year 2021, opioid use for pain was more frequent among First Nations People living outside versus within First Nations communities (118.2 vs. 91.2 per 1000, respectively) and among females relative to males (124.6 and 93.9 per 1000, respectively). Concurrent prescription benzodiazepine use among First Nations People receiving a prescription opioid for pain decreased from 20.9% in Q2 2013 to 16.7% in Q4 2021. In stratified analyses, concurrent use was more prevalent among females, adults aged ≥ 65 years, and First Nations People living outside First Nations communities.

**Conclusion:**

Opioid analgesic prescribing patterns for First Nations People living in Ontario indicate a decrease in both overall prescribing rates and concurrent benzodiazepine use.

## Résumé

**Objectifs:**

Investiguer les tendances de délivrance des opioïdes contre la douleur et des traitements par benzodiazépines concomitants et les caractéristiques des personnes des Premières Nations de l’Ontario auxquelles sont délivrés un opioïde contre la douleur et un traitement par benzodiazépine concomitant.

**Méthodes:**

Nous avons mené une étude transversale sérielle populationnelle par trimestre des personnes des Premières Nations inscrites (reconnues) en Ontario auxquelles ont été délivrés un opioïde contre la douleur entre le 1er avril 2013 et le 31 décembre 2021. Nous avons déclaré les tendances trimestrielles de la prévalence et de l’incidence de la délivrance d’opioïdes (taux pour 1000 personnes) et la prévalence de l’emploi concomitant de benzodiazépines chez les sujets recevant des opioïdes contre la douleur. La dernière année de l’étude (2021), nous avons stratifié les taux par âge, par lieu de résidence (à l’intérieur ou à l’extérieur des communautés des Premières nations) et par sexe.

**Résultats:**

Entre 2013 et 2021, le taux trimestriel de délivrance d’opioïdes contre la douleur a diminué de 25% chez les personnes des Premières Nations en Ontario, passant de 74,7 à 56 pour 1000. Dans nos analyses stratifiées pour l’année 2021, l’emploi d’opioïdes contre la douleur était plus fréquent chez les personnes des Premières Nations vivant hors des communautés des Premières nations que chez celles vivant dans ces communautés (118,2 contre 91,2 pour 1000, respectivement), et plus fréquent chez les femmes que chez les hommes (124,6 et 93,9 pour 1000, respectivement). L’emploi concomitant de benzodiazépines d’ordonnance chez les personnes des Premières Nations recevant un opioïde d’ordonnance contre la douleur a diminué, passant de 20,9% au deuxième trimestre de 2013 à 16,7% au quatrième trimestre de 2021. Dans nos analyses stratifiées, l’emploi concomitant était plus prévalent chez les femmes, les adultes de ≥ 65 ans et les sujets vivant hors des communautés des Premières nations.

**Conclusion:**

Les tendances de la prescription d’opioïdes analgésiques aux personnes des Premières Nations vivant en Ontario affichent une diminution à la fois des taux de prescription globaux et de l’emploi concomitant des benzodiazépines.

## Introduction

Opioid analgesics are commonly dispensed for the treatment of pain. To mitigate the risks of iatrogenic opioid-related harm, prescribing guidelines recommend reserving these drugs for cases where other options have been ineffective, using the lowest effective dose and avoiding concurrent use of other respiratory depressants, such as benzodiazepines (Busse et al., 2017). In Ontario, use of prescription opioids for pain declined following publication of these guidelines, decreasing from 122.8 per 1000 individuals in 2013 to 88.8 per 1000 in 2021 (Ontario Drug Policy Research Network, 2024).

Yet, despite these data, little is known of the dispensing trends and characteristics of individuals receiving opioids for pain among First Nations People living in Ontario. This is important because First Nations People have been disproportionately impacted by the opioid toxicity crisis in Canada and have a higher incidence of pain and pain-related disability relative to the non-First Nations population (Chiefs of Ontario & Ontario Drug Policy Research Network, 2022; Health Canada, 2020). Moreover, First Nations People face systemic barriers when accessing pain treatment, including clinician dismissal of symptoms and further marginalization through healthcare when seeking pain management (Health Canada, 2020). In order to understand the changing dynamics of opioid use among First Nations People and inform health policies and interventions, First Nations leaders and researchers have prioritized a need for research evaluating prescription opioid use trends. Accordingly, we investigated prescription opioid dispensing trends and the characteristics of First Nations People in Ontario dispensed an opioid for pain and concurrent benzodiazepine treatment between 2013 and 2021.

## Methods

This study was led by the Chiefs of Ontario (COO) in collaboration with the Ontario Drug Policy Research Network (ODPRN). The research is mandated by Ontario First Nations leadership under Resolution 20/18 Prescription Opioid Surveillance. Research staff members from COO, the Circle of Lived Experience Advisory Committee, and the Opioid Surveillance Steering Committee, representing First Nations communities across Ontario, provided advice and knowledge on the analysis plan and data interpretation. The research adheres to the First Nations principles of OCAP® (ownership, control, access, and possession) and the Consolidated Criteria for Strengthening Reporting of Health Research Involving Indigenous Peoples (CONSIDER) framework (Huria et al., 2019).

### Setting and data sources

We conducted a population-based serial cross-sectional study by quarter of all registered (Status) First Nations People in Ontario who were dispensed an opioid for pain between April 1, 2013, and December 31, 2021. We used administrative health data at ICES, an independent, non-profit research institute whose legal status under Ontario’s health information privacy law allows it to collect and analyze healthcare and demographic data, without consent, for health system evaluation and improvement. We used the Indian Registry System (IRS) database to identify all registered (Status) First Nations People in Ontario, which includes people who are eligible and have registered for ‘Indian Status’ under the ‘Indian Act’. We acknowledge that language used in this legislation, such as ‘Indian’ to describe First Nations People, is outdated, offensive, and rooted in colonialism. We use such terminology only when needed to reflect source documents.

We used the Registered Person Database (RPDB), a registry of all Ontarians eligible for publicly funded health insurance, to ascertain the demographic and geographic characteristics of all individuals. We used the Narcotics Monitoring System (NMS) database, which contains comprehensive records of all prescriptions for controlled substances dispensed from community pharmacies, to identify the dispensing of opioids and benzodiazepines. For each year of the study period, residence within and outside of First Nations communities was determined using the address provided at healthcare encounters. If a person did not have a healthcare encounter in the year of interest, their postal code was obtained from their health card. All datasets included in this analysis were linked using unique encoded identifiers and analyzed at ICES in Toronto, Ontario (https://www.ices.on.ca). The use of the data in this project is authorized under Sect. 45 of Ontario’s Personal Health Information Protection Act (PHIPA) and does not require review by a Research Ethics Board. Furthermore, use of these data was approved by the COO First Nations Data Governance Committee. To minimize the risk of re-identification, small counts (*N* < 6) were not reported.

### Cohort definition and exposure

We identified all First Nations People who received at least one prescription for an opioid indicated for pain. We defined an individual’s index date as the date of the first prescription claim for an opioid for pain in each quarter. Each quarter, we defined prevalent users as those who filled a prescription for an opioid analgesic, overall and stratified according to residence within or outside of a First Nation community. New users of prescription opioids (incident users) were defined as people who had not received a prescription opioid for pain, cough, or opioid agonist therapy (OAT) in the previous 365 days prior to the index date. We calculated the quarterly rates of incident and prevalent opioid use per 1000 First Nations People and reported quarterly trends in dispensing between April 1, 2013 (Q2 2013) and December 31, 2021 (Q4 2021). We used drug and product identification numbers to identify prescription opioids primarily indicated for pain, included all available dosage forms and formulations, and excluded opioid products indicated as anti-diarrheal, cough suppressants, and OAT to ensure our cohort was limited to individuals who dispensed opioids for pain. Lastly, we reported the quarterly prevalence of concurrent benzodiazepine use among individuals prescribed opioids for pain. We defined this as any individual who had overlapping periods of use for both classes of medications, using the days’ supply indicated on each prescription claim.

### Statistical analysis

For analyses of quarterly trends, we reported the relative percentage change over the study period overall and stratified by location of residence. In the final year of the study (2021), we reported the population-adjusted rates of prescription opioid use for pain (incidence and prevalence) among First Nations People in Ontario overall and across several strata, including place of residence (within or outside First Nations communities), age (< 25 years, 25–44 years, 45–64 years, and ≥ 65 years), and sex (female and male). We used the total population of First Nations People residing in Ontario in the time period of interest (i.e., quarter or year), overall and in each strata to define population denominators. Lastly, we described the distribution of opioid types dispensed among prevalent recipients in the first year of the study period (April 1, 2013 to March 31, 2014) versus the last year of the study period (January 1, 2021 to December 31, 2021), and used chi-square tests to compare proportions. All analyses were performed at ICES using SAS version 9.4.

## Results

### Trends in prescribing over time

**Fig. 1:**
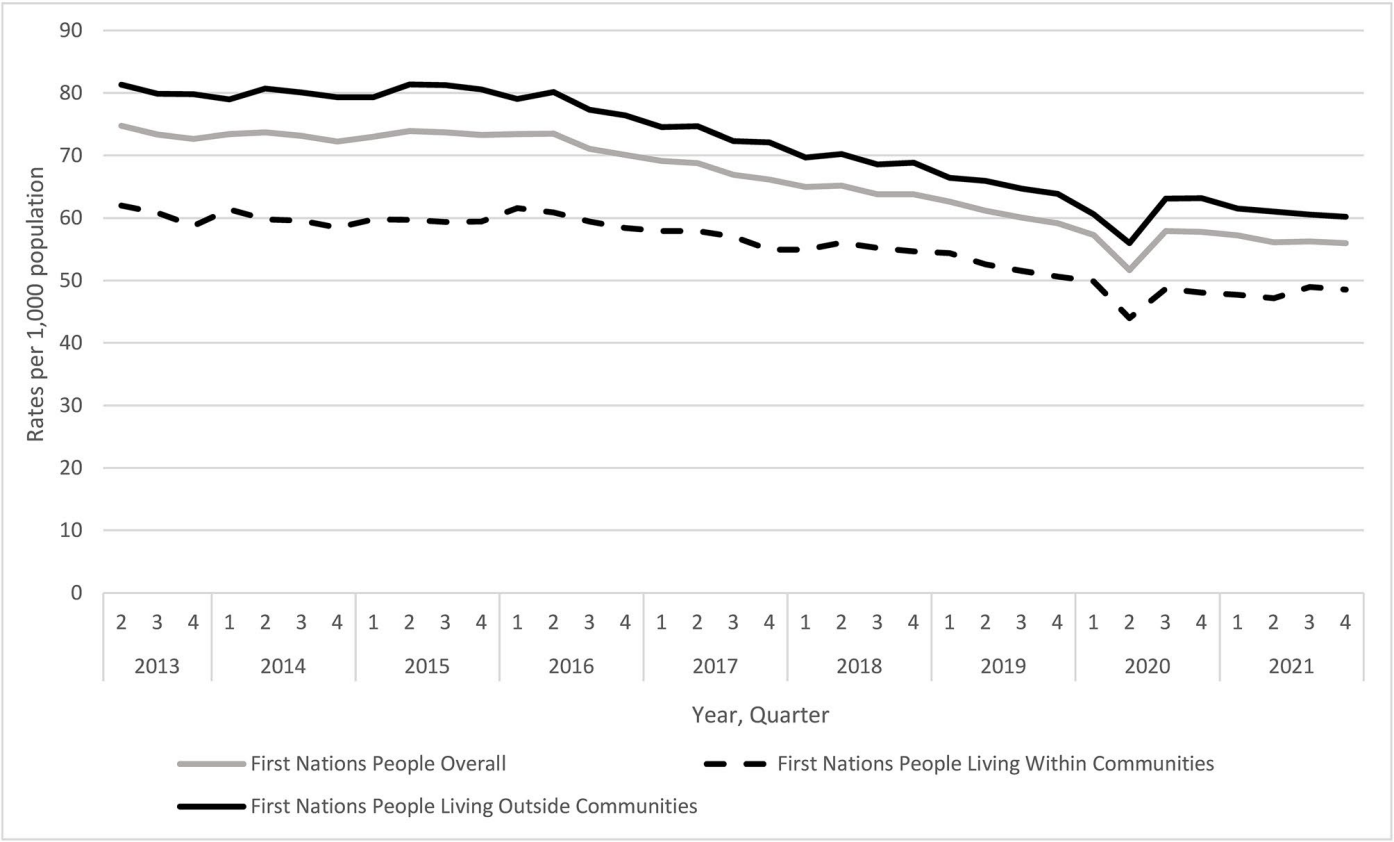
Quarterly trends in the prescribing of opioids for pain (rates per 1000) among First Nations People in Ontario between Q2 2013 and Q4 2021

**Fig. 2:**
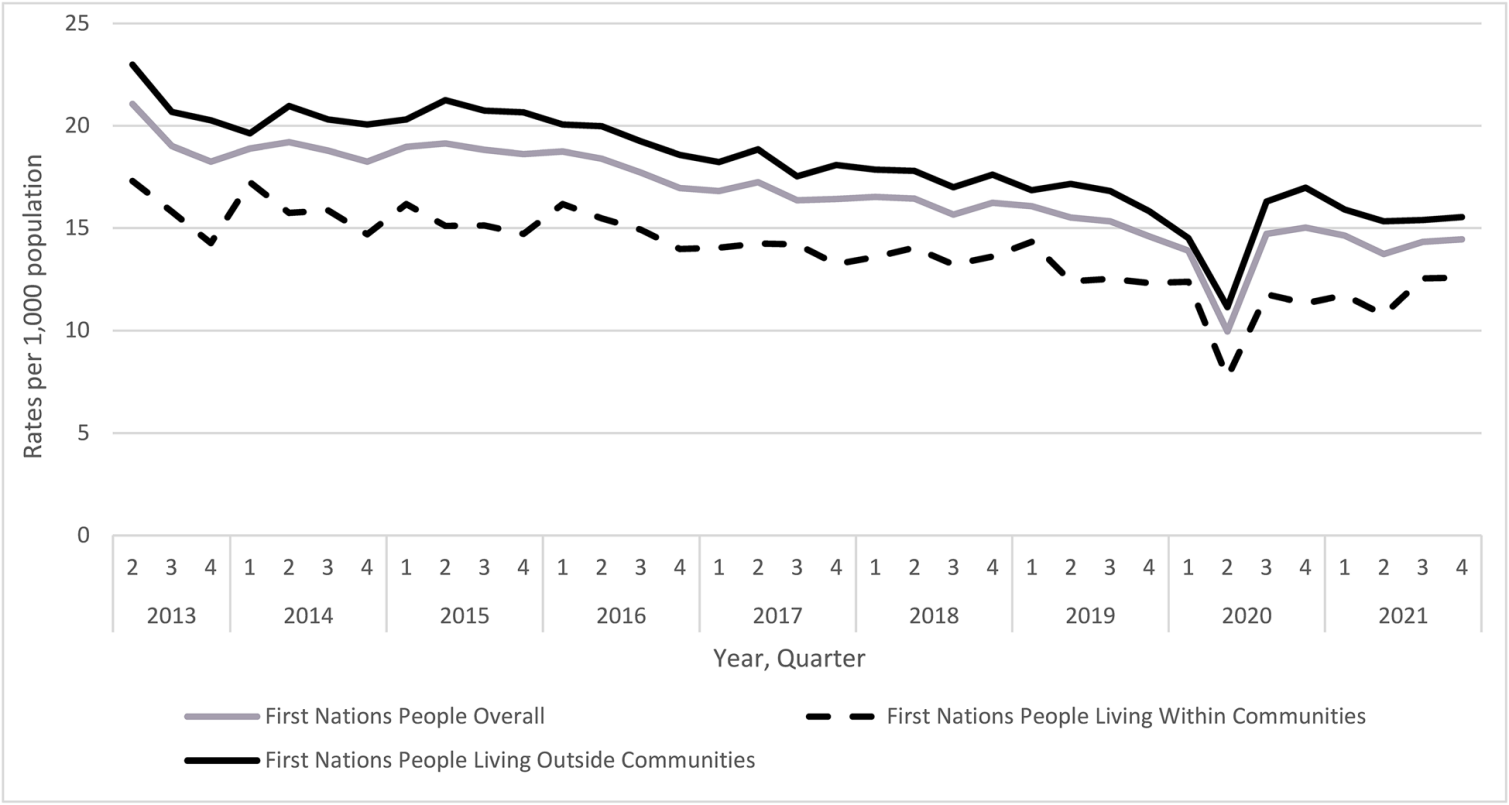
Quarterly trends in the initiation of opioids for pain (rates per 1000) among First Nations People in Ontario between Q2 2013 and Q4 2021

Over the eight-and-a-half-year study period, we observed a 25.0% decrease in the overall rate of dispensing of opioids for pain among First Nations People in Ontario (74.7 vs. 56.0 per 1000 First Nations People in Q2 2013 and Q4 2021, respectively) (Fig. 1). A temporary decrease in opioid dispensing was observed in the second quarter of 2020 (51.1 per 1000), corresponding with the onset of the COVID-19 pandemic. Incident use of opioids for pain declined by 31.3% over the study period (21.1 vs. 14.5 per 1000 people in Q2 2013 and Q4 2021, respectively) (Fig. 2). In analyses stratified by location of residence, opioid dispensing rates declined similarly over time, but were higher among First Nations People living outside First Nations communities compared to individuals living within First Nations communities (Q4 2021: 60.2 and 48.6 per 1000, respectively).

### Characteristics of individuals prescribed opioids for pain

In 2021, 18,208 First Nations People in Ontario (109.1 per 1000 people) were prescribed an opioid for pain, and 9506 newly initiated prescription opioids for pain treatment (56.9 per 1000 people) (Table 1). The prevalence of prescribing increased with age, with the highest rate observed among adults aged 65 years or above (208.6 per 1000 persons). Opioid use was more prevalent among females relative to males (124.6 and 93.9 per 1000, respectively) and among First Nations People living outside relative to within First Nations communities (118.2 and 91.2 per 1000, respectively). Patterns of incident opioid use were similar, with a higher rate among females compared to males (65.4 and 48.7 per 1000, respectively), older adults ≥ 65 years (85.6 per 1000 compared to 40.1 per 1000 among young people < 25 years), and First Nations People living outside compared to people living within First Nations communities (62.1 per 1000 vs. 47.5 per 1000 First Nations People, respectively). We observed significant shifts in the types of opioids commonly dispensed over time. Specifically, when comparing the first and last years of the study period, we observed decreased dispensing of codeine combination products (60.6% vs. 46.4%; *p* < 0.0001) and oxycodone combination products (29.1% vs. 20.8%; *p* < 0.0001), while hydromorphone dispensing increased (10.6% vs. 25.5%; *p* < 0.0001) (Table 2).

**Table 1:** Characteristics of First Nations People dispensed opioids for pain, in 2021

	Overall prescription use, ***N*** (rate per 1000) (Prevalence)	New prescription use, ***N*** (rate per 1000) (Incidence)	Individuals receiving an opioid for pain concurrently prescribed a benzodiazepine, ***N*** (%)
Overall	18,208 (109.1)	9506 (56.9)	2573 (14.1%)
Age (years) < 25 25–44 45–64 ≥ 65	1969 (44.2) 4779 (83.0) 7637 (164.0) 3823 (208.6)	1785 (40.1) 3002 (52.2) 3151 (67.7) 1568 (85.6)	52 (2.6%) 488 (10.2%) 1293 (16.9%) 740 (19.4%)
Sex Female Male	10,279 (124.6) 7929 (93.9)	5396 (65.4) 4110 (48.7)	1637 (15.9%) 936 (11.8%)
Location of residence Within community Outside of community	4887 (91.2) 13,172 (118.2)	2546 (47.5) 6917 (62.1)	526 (10.8%) 1979 (15.0%)

**Table 2:** Annual distribution of First Nations People receiving opioids for pain in the first year versus the last year of the study period, by type of opioid dispensed

Type of opioid dispensed^a^, *N* (%)	April 2013–March 2014	January–December 2021	*P*-value
Codeine	891 (3.5%)	479 (2.6%)	< 0.0001
Codeine combination products^b^	15,440 (60.6%)	8439 (46.4%)	< 0.0001
Fentanyl	533 (2.1%)	103 (0.6%)	< 0.0001
Hydromorphone	2692 (10.6%)	4640 (25.5%)	< 0.0001
Morphine	2622 (10.3%)	2252 (12.4%)	< 0.0001
Oxycodone	934 (3.7%)	606 (3.3%)	0.0590
Oxycodone combination products^c^	7401 (29.1%)	3708 (20.8%)	< 0.0001
Tramadol	1865 (7.3%)	1781 (9.8%)	< 0.0001
Other^d^	442 (1.7%)	181 (1.0%)	< 0.0001

^a^Percentage of prevalent opioid recipients dispensed each opioid type. The denominator is 25,476 and 18,208 First Nations People in Ontario dispensed an opioid for pain in April 2013–March 2014 and January–December 2021, respectively

^b^For example codeine and acetaminophen

^c^For example oxycodone and acetaminophen

^d^Includes buprenorphine, methadone, meperidine, and other opioid types

### Co-prescribing of benzodiazepines

**Fig. 3:**
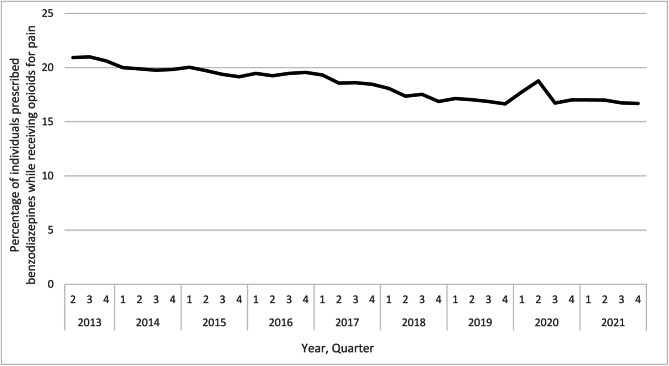
Quarterly trends in concurrent benzodiazepine prescribing among First Nations People receiving an opioid for pain from Q2 2013 to Q4 2021

During the study period, we observed a 20.1% decline in the prevalence of concurrent benzodiazepine prescribing among First Nations People in Ontario receiving an opioid (20.9% in Q2 2013 vs. 16.7% in Q4 2021; Fig. 3). In stratified analyses, we found that in 2021, concurrent prescription opioid and benzodiazepine use was more prevalent among females (15.9%) compared to males (11.8%), adults aged ≥ 65 years (19.4%) compared to those aged < 25 years (2.6%), and First Nations People living outside First Nations communities (15.0%) compared to people living within First Nations communities (10.8%).

## Discussion

In our retrospective population-based study, we observed a 25.0% decline in quarterly rates of opioid prescriptions dispensed for pain and a 20.1% decrease in concurrent opioid and benzodiazepine dispensing among First Nations People in Ontario over the study period. Additionally, we observed a rapid, short-term decline in opioid dispensing rates at the start of 2020, coinciding with the beginning of the COVID-19 pandemic and associated disruptions to medication dispensing, which quickly resolved and returned to previous pre-pandemic trends. Moreover, there were notable shifts in the types of opioids for pain dispensed over time, including increased hydromorphone dispensing and decreased dispensing of codeine- and oxycodone-containing products. Overall, our findings suggest that changes in opioid prescribing for pain have occurred that were consistent with those observed in the entire Ontario population (Gomes et al., 2024), and which may reflect the publication of updated guidelines in 2017 (Busse et al., 2017).

Our findings have important implications for the health of First Nations People. Specifically, despite the decline in co-prescribing of benzodiazepines and opioids, 14.1% of First Nations People prescribed an opioid in 2021 were still at risk of potential harms from concurrent use. Moreover, the combined use of these medications was higher among women, adults aged ≥ 65 years, and people living outside First Nations communities. While the use of opioids and benzodiazepines together may sometimes be indicated, further research is required to understand the appropriateness of patterns of concurrent use and whether rates of combined use observed in this study are influenced by a lack of access to programs and services needed to help address the trauma and related mental health challenges resulting from the intergenerational impacts of colonization among First Nations People (Bombay et al., 2009; Brockie et al., 2015; Konkolÿ Thege et al., 2017; Spillane et al., 2023). These data are also necessary prerequisites for the development and implementation of culturally informed benzodiazepine and opioid de-prescribing initiatives.

Our findings are comparable with research from other provinces, with decreases in opioid dispensing among First Nations individuals reported in Alberta between 2016 and 2021 (Alberta First Nations Information Governance Centre & Government of Alberta, 2024). When compared to overall population data from Ontario investigating opioid prescribing trends in 2021, our study shows a slightly higher rate of prescribing among First Nations People (88.8 and 109.1 per 1000, respectively) and similar prescription opioid initiation rates (both approximately 60.0 per 1000) (Ontario Drug Policy Research Network, 2024). Similarly, higher opioid prescribing rates were reported among First Nations People living in Alberta and Manitoba compared to non-First Nations populations (Alberta First Nations Information Governance Centre & Government of Alberta, 2024; Katz et al., 2019). These findings may reflect a higher prevalence of self-reported moderate or severe pain among First Nations individuals and disparities in available pain treatment modalities such as physiotherapy, occupational therapy, and cognitive behavioral therapy for First Nations People. Moreover, the intergenerational impacts of colonialism, social inequities, and the historic experiences of systemic discrimination in healthcare influence the experience of pain among First Nations individuals and perpetuate experiences of stigma and judgement (Smye et al., 2023), when accessing healthcare for pain (Nelson & Wilson, 2018). As a result, it is important that healthcare providers treating First Nations People for pain receive adequate training in culturally appropriate and trauma-informed care (Bailey et al., 2023; Browne et al., 2021; Elder Dumont, National Native Addictions Partnership Foundation, & CIHR, 2014), and that there be equitable investment in pain assessment and treatment modalities that are supported by best practice standards for First Nations People across the country.

We observed higher rates of opioid use for pain among women and older adults (aged ≥ 65 years), a pattern similar to previous research (Ontario Drug Policy Research Network, 2024; Serdarevic et al., 2017). This difference may reflect sex- and age-related differences in chronic pain prevalence (Meana et al., 2004) (Domenichiello & Ramsden, 2019). We also observed that a larger proportion of First Nations People living outside First Nations communities received a prescription for an opioid for pain compared to individuals living within First Nations communities. This may highlight the differences in access to culturally appropriate, non-pharmaceutical options available to people within First Nations communities, as well as potential barriers to accessing healthcare for First Nations communities situated in more remote regions.

Our study has some limitations; we identified First Nations People using the Indian Registry System database, which includes people who are eligible and registered for ‘Indian Status’ under the *Indian Act.* Therefore, anyone who is not a registered First Nations person as identified in the Indian Registry System is not included in this analysis. In our analysis, some of the slow-release oral morphine (SROM) and immediate-release hydromorphone products captured may be used in the treatment of opioid use disorder or within safer opioid supply programs, although it is anticipated that this prevalence will be low. For benzodiazepine prescribing, we were unable to determine the appropriateness of treatment as we did not capture the indication for treatment (e.g., seizure disorder) or duration of treatment. Due to the absence of clinical indication of opioid use in prescribing claims data, we were not able to capture intended use of opioids (i.e., for acute vs. chronic pain); however, we relied on drug identification numbers to identify prescription opioids indicated for pain dispensed over the study period. Additionally, we may have misclassified dispensing of immediate-release hydromorphone and slow-release oral morphine, which are clinically indicated for pain but increasingly being prescribed for additional indications (i.e., as OAT and safer opioid supply, respectively). However, we expect that the influence on trend will be minimal given studies that have pointed to small numbers of people prescribed these medications for these non-pain indications throughout our study period (Ontario Drug Policy Research Network, 2024; Young et al., 2022). Finally, we did not measure duration of opioid treatment over time and were unable to determine whether decreasing prevalence of opioid use was associated with appropriate treatment tapering. Past research has found that rapid opioid dose tapering can lead people to access the toxic unregulated opioid supply and worsen risks of harms for people previously using pharmaceutical opioids. Therefore, future work is needed to understand patterns of opioid discontinuation among First Nations People.

## Conclusion

Overall, this study suggests considerable changes in opioid dispensing patterns among First Nations People living in Ontario since the introduction of new clinical guidelines for the management of chronic non-cancer pain, with decreases in overall prescribing and concurrent benzodiazepine use. Future work is needed to support community-directed efforts to balance adequate pain management using pharmaceuticals with traditional and land-based therapies among First Nations People across Ontario (Radu, 2018; Sommerfeld et al., 2019).

### Contributions to knowledge

What does this study add to existing knowledge?

- This study sheds light on opioid prescribing trends and characteristics among First Nations People in Ontario, a group disproportionately affected by the opioid crisis. It addresses the gap in knowledge regarding the dispensing trends and characteristics of individuals receiving opioids for pain within this population.
- Our results demonstrate a significant decline in opioid prescriptions for pain and concurrent opioid and benzodiazepine use among First Nations People in Ontario. Lower prescribing rates within First Nations communities could suggest differences in access to culturally appropriate, non-pharmaceutical options and barriers to healthcare in remote areas.

What are the key implications for public health interventions, practice or policy?

- Public health interventions should adopt a multifaceted approach to address the risks associated with concurrent opioid and benzodiazepine use among First Nations People, particularly among high-risk groups such as women and older adults. This includes implementing policies and safety guidelines promoting the appropriate use of these medications, supporting culturally informed pain management practices, ensuring equitable access to pharmacologic and non-pharmacologic treatments, and addressing barriers to healthcare access for First Nations People, especially those in remote areas. Implementing these strategies can enhance access to safe, effective, culturally responsive pain management options for First Nations People, leading to better health outcomes.

## Data Availability

The dataset from this study is held securely in coded form at ICES. While legal data-sharing agreements between ICES and data providers (e.g., healthcare organizations and government) prohibit ICES from making the dataset publicly available, access may be granted to those who meet pre-specified criteria for confidential access, available at www.ices.on.ca/DAS (email: das@ices.on.ca).

## Code availability

The full dataset creation plan and underlying analytic code are available from the authors upon request, understanding that the computer programs may rely upon coding templates or macros that are unique to ICES and are therefore either inaccessible or may require modification.
